# UV–O_3_ treated annealing-free cerium oxide as electron transport layers in flexible planar perovskite solar cells†

**DOI:** 10.1039/d0na00367k

**Published:** 2020-07-23

**Authors:** Aiying Pang, Jinlong Li, Xiao-Feng Wei, Zhi-Wu Ruan, Ming Yang, Zhong-Ning Chen

**Affiliations:** State Key Laboratory of Structural Chemistry, Fujian Institute of Research on the Structure of Matter, Chinese Academy of Sciences Fuzhou Fujian 350002 China; College of Chemistry and Materials, Fujian Normal University Fuzhou Fujian 350007 China; College of Chemistry, Fuzhou University Fuzhou Fujian 350002 China; National Engineering Research Center of Chemical Fertilizer Catalyst, School of Chemical Engineering, Fuzhou University Fujian 350002 China

## Abstract

Fabricating electron transport layers at low temperatures is challenging but highly desired in the field of flexible perovskite solar cells (f-PSCs). In this study, highly uniform cerium oxide (CeO_*x*_) films prepared by the UV–O_3_ treatment have been successfully applied as the electron transport layer (ETL) in methylammonium lead halide (CH_3_NH_3_PbI_3_) perovskite-based f-PSCs. Under AM 1.5 G sunlight with 100 mW cm^−2^, these cells exhibited an open-circuit voltage (*V*_oc_) of 0.98 V, a short-circuit current density (*J*_sc_) of 19.42 mA cm^−2^, a fill factor (FF) of 0.72 and power conversion efficiency (PCE) of 14.63%. The PCE was much higher than that of the control planar CeO_*x*_ ETL (PCE ∼ 9.08%) prepared at a low temperature (80 °C) without the UV–O_3_ treatment, and this was ascribed to the improved CeO_*x*_ film, enhanced light absorption and suppressed charge recombination. The cells that bend at 15 mm of radius showed excellent stability with less than 10% reduction in PCE after 500 cycles of repeated bending at ambient temperature. The charge-transmission kinetic parameters and long-term stability of the CeO_*x*_-based f-PSCs were analyzed as well.

## Abstract 2

UV–O_3_ treated annealing-free cerium oxide (CeO_*x*_) is used as the electron transport layers of flexible planar perovskite solar cells to achieve the highest PCE of 14.63%. The devices retained *ca.* 90% efficiency of the initial value after recovery from 500 bending cycles at *r* = 15 mm.

## Graphical abstract

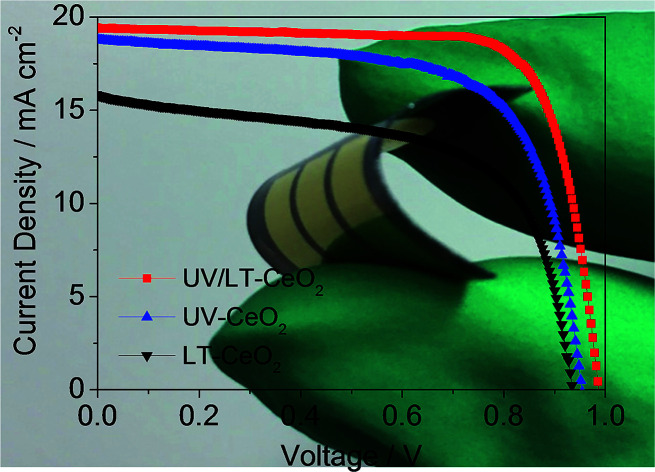

## 1. Introduction

Perovskite solar cells (PSCs) are very promising devices in photovoltaic applications due to their impressive efficiency increasing and efficient energy harvesting. Most academic and industrial researches have been focused on PSCs immobilized on rigid substrates.^1,2^ However, rigid glass substrates are generally fragile and difficult for scale-up utilization. Incontrovertibly, the flexible perovskite solar cells (f-PSCs) hinged on plastic substrates, which are readily bendable and expediently portable, are suitable for wearable electricity-generating devices and building integrated photovoltaics. In fact, relevant advancements in n–i–p planar or p–i–n cells on plastic substrates achieved the highest reported power conversion efficiency (PCE) of 20.01%.^3–5^ Apart from the high PCE, this new photovoltaic technology can be achieved using low temperatures (<150 °C) to convert the perovskite and electron transport layer (ETL) precursors into their final semiconducting forms, which are then immobilized onto transparent plastic substrates, such as polyethylene naphthalate (PEN) and polyethylene terephthalate (PET).

Nevertheless, some new obstacles have been found in f-PSCs. Titanium oxide (TiO_2_), the most widely used material in the compact layer and mesoporous layer (collectively called ETL) in the regular architecture of PSCs, typically sustains high-temperature treatment (500 °C).^6,7^ This is incompatible with plastic substrates, whose processing temperature must be controlled below 150 °C. Till now, only one study has reported the use of mesoporous TiO_2_ in f-PSCs made of polymer films,^8^ whereas it is more regularly used on metal-foil substrates that can endure high temperatures.^9^ In addition, Nb-doped TiO_2_ ETLs fabricated *via* a low-temperature (<50 °C) UV process exhibited the best PCE of 16.01% when used in n–i–p f-PSCs.^10^ ETLs generated from Ti-based metal–organic framework nanoparticles at ambient temperature have been found suitable for charge injection and transfer from the perovskite to the electrodes.^11^ Although a TiO_*x*_-based ETL prepared through a low-temperature (<100 °C) solution process has achieved up to 17.6% of PCE, TiO_2_-based PSCs can be easily destroyed by UV light.^12^ This intrinsic property inhibits the commercial applications of TiO_2_-based PSCs.^13,14^ ZnO is considered as the most promising material for f-PSCs because it has the same conduction band at 3.2 eV as that of TiO_2_, a higher electron mobility of 115–155 cm^2^ V^−1^ s^−1^ and especially an easier low-temperature synthetic process.^15,16^ When a mesoporous layer grown from ZnO nanorods using a chemical bath was first implemented in f-PSCs, it showed a PCE of 5.0% on PET/ITO, showing a great prospect.^16^ The ink-dispersed ZnO nanoparticles fabricated by the spin-coating process are widely employed as ETLs in inverted devices.^17,18^ The planar solar cell configuration of PEN/ITO/ZnO/CH_3_NH_3_PbI_3_/PTAA/Au delivered the highest efficiency of 15.6% at the time of publication.^19^ However, the hydroxide groups in the ZnO nanoparticles are very susceptible to acid and base solutions, which is detrimental to the long-term stability of f-PSCs, leading to the degradation of the perovskite layer.^20,21^ Alternatively, SnO_2_ is a UV- and chemical-stable material for PSCs with a larger bandgap and higher electron mobility than TiO_2_ and ZnO.^22,23^ The f-PSCs based on solution-processing SnO_2_ ETLs could achieve a PCE of 17.21%; however, the processing temperature of up to 180 °C is far greater than the maximum tolerable temperature (150 °C) of the flexible substrates.^24^ On the other hand, though SnO_*x*_ prepared by atomic layer deposition satisfies the temperature requirements and exhibits good electron selectivity, the fabrication process inhibits its application in roll-to-roll technology and commercial appeal.^25,26^ Therefore, it is necessary to search for new type ETLs with processing temperatures under 150 °C and enhance long-term stability.

Cerium oxide (CeO_*x*_) has a wide bandgap with an effective dielectric constant, good transparency, sufficient ionic conductivity, and high-temperature stability, and has been used in various optoelectronic applications, such as light absorber in organic-dye-free solar cells, photoanode in-dye sensitized solar cells, and antireflection coating in silicon solar cells.^27,28^ It is worth noting that CeO_*x*_ used recently in PSCs exhibited better photoelectric properties under 6,6-phenyl-C_61_-butyric acid methyl ester modification or when employed as a dense diffusion barrier in the p–i–n structure.^29,30^ In this study, we report CeO_*x*_ aqueous sol–gel as a precursor for ETL preparation by the UV–O_3_ treatment for use in flexible n–i–p planar PSCs in the quest for a low-cost and scale-up manufacturing technology. The compact CeO_*x*_ layers treated by low-temperature annealing (LT-CeO_*x*_), ultraviolet–ozone (UV–O_3_) (UV-CeO_*x*_), and a co-processing treatment (UV/LT-CeO_*x*_) were characterized by studying the hydrophilicity contact angle and surface behavior. In order to verify the difference of device ETLs, photoelectric tests of the cells with, as well as without UV–O_3_ treatment, were carried out. As demonstrated experimentally, the UV–O_3_-treatment of the compact CeO_*x*_ layer imparted a positive effect on the cell performance, improving the efficiency from 9.08% to over 14%.

## 2. Experimental section

### 2.1 CeO_*x*_ sol–gel solution preparation

CeO_*x*_ NCs were synthesized by a modified procedure described in the literature.^31^ 6.0 g of Ce(NO_3_)_3_·6H_2_O (99%, Aldrich) was added to 70 mL distilled water with stirring till it was sufficiently dissolved. An NH_4_OH (NH_3_ content 28–30%, Aldrich, ACS reagent) solution was then added dropwise until a pH of 10 was reached. Then, cerium nitrate completely converted to cerium hydroxide and precipitated, which was separated by centrifugation and washed with deionized water repeatedly. Then, it was dissolved in 140 mL of a 0.1 M urea aqueous solution and adjusted to pH 2 by the addition of dilute hydrochloric acid (10%). The concentration of the obtained solution was assessed as 82 mM by calculating its xerogels at 600 °C for 2 h. The obtained CeO_*x*_ sol–gel solution was stored at room temperature to prepare ETLs.

### 2.2 CH_3_NH_3_I preparation

CH_3_NH_3_I was synthesized and purified using a method described in the literature.^32^ 10 mL hydroiodic acid (57 wt%) was added dropwise with stirring to a 33 wt% methanol solution of methylamine (24 mL) at the temperature of 0 °C. After stirring for 2 h in an N_2_ atmosphere, the obtained liquid–solid mixture was evaporated by a liquid-volatile separation process at 50 °C for 1 h to remove volatile substances. Then, the resultant crystal was permeated with three 70 mL portions of absolute diethyl ether and dried at 60 °C in a vacuum oven for 12 h to afford the desired product with high purity.

### 2.3 Device fabrication

ITO-coated flexible substrates (polyethylene naphthalate, PEN) were sequentially cleaned by acetone and ethanol for 15 min and then by UV–ozone (UV–O_3_) treatment for another 15 min. The cleaned flexible substrates were spin-coated with the CeO_*x*_ colloid for 30 s at 2000 rpm. The UV-CeO_*x*_ ETLs were obtained after drying at 80 °C for 10 min and exposure to UV–O_3_ for 15 min. The LT-CeO_*x*_ ETLs were obtained just by drying at 80 °C for 20 min. Identical processes and treating conditions were repeated for adjusting the thickness of the ETLs. After that, the ETLs were infiltrated with the perovskite precursor solution by the spin-coating process in a glovebox under a nitrogen atmosphere. The perovskite precursor solution was prepared using a stoichiometric amount of CH_3_NH_3_PbI_3_ in 1.2 M DMSO with lead iodide and methyl ammonium iodide at a molar ratio of 1 : 1.^32^ The spin coating procedure was firstly performed at 6000 rpm for 20 s and then for 10 s by gently dropping chlorobenzene on the spinning substrate with a micropipette. Then, the substrate was heated at 100 °C for 1 h on a hot plate in the glovebox. After they were cooled to 25 °C, the spiro-OMeTAD solution (60 μL) was spin-coated on the CH_3_NH_3_PbI_3_ layer at 3000 rpm for 20 s to be used as the hole transparent layer. Spiro-OMeTAD was dissolved in chlorobenzene at 72 mg mL^−1^, to which 28.8 μL of 4-*tert*-butyl pyridine and 14.4 μL of lithium bis(trifluoromethanesulfonyl)-imide (Li-TFSI) solution (520 mg Li-TFSI in 1 mL acetonitrile) were added. Finally, an 80 nm gold layer was deposited on the coated spiro-OMeTAD film at ∼10^−6^ bar *via* thermal evaporation.

### 2.4 Characterization

The XRD patterns were measured on a PANalytical X'Pert spectrometer using Co Kα radiation (*λ* = 1.78897 Å), and the data were converted to Cu Kα data. SEM was performed on a Hitachi S4800 instrument. TEM and SAED pattern images were recorded on a Tecnai G2 F20 (FEI) with an accelerated photo-voltage of 200 kV. The FT-IR spectra were obtained on a PerkinElmer Spectrum 2000. The survey scans were recorded using monochromatic Al Kα irradiation, 1 eV steps and an 80 eV analyser. The transmittance spectra and UV-vis spectra were measured on a Lambda-9 (PerkinElmer) spectrometer. The steady-state photoluminescence (PL) and time-resolved photoluminescence (TRPL) decay spectra of CH_3_NH_3_PbI_3_ coated on the LT-CeO_*x*_, LT-CeO_*x*_, and UV/LT-CeO_*x*_ ETLs were recorded using an Edinburgh FLS920 instrument with a picosecond-pulsed diode laser at 375 nm. The UPS for the LT-CeO_*x*_, LT-CeO_*x*_, and UV/LT-CeO_*x*_ ETLs were measured using ESCALAB 250Xi (Thermo Fisher) under a background pressure of 5.0 × 10^−7^ Pa.

The photovoltaic performance in terms of *J*–*V* characteristics was measured on a solar simulator (Sol3A Class AAA, Oriel Instruments, Stratford, CT, USA) and a Keithley 2440 source measurement unit (Keithley Instruments Inc., Cleveland, OH, USA) under 1.5 air mass (AM) and 1 sun (100 mW cm^−2^) condition. The 1-sunlight intensity level was calibrated using a standard Si reference cell certified by the Newport Corporation. All the devices were measured in a light-tight sample holder with an active area of 0.12 cm^2^ for each cell and fixed using an aperture mask. IPCE was measured using a quantum efficiency measurement system (QEX10, PV Measurements, Inc.) in the wavelength range of 300 to 850 nm. EIS was recorded using a potentiostat (IM-6, Zahner) in the frequency range of 0.1 to 100 kHz by applying the *V*_oc_ values derived from the *J*–*V* tests and under dark conditions.

## 3. Results and discussion

**Fig. 1:**
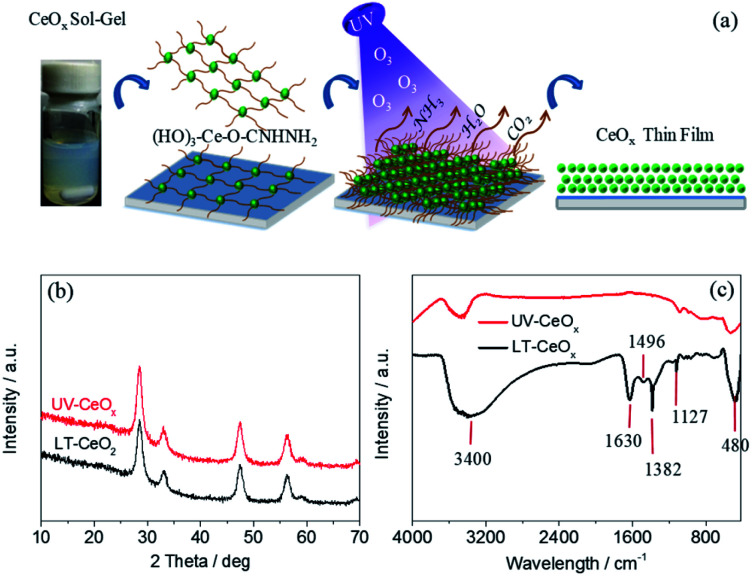
(a) Schematic illustration of the UV–O_3_ process for the preparation of CeO_*x*_ thin films. (b) XRD spectra and (c) FT-IR spectra of the CeO_*x*_ thin films before (LT-CeO_*x*_) and after (UV-CeO_*x*_) UV–O_3_ treatment.

The ligand-capped CeO_*x*_ sol–gel solution prepared using a 0.1 M aqueous urea solution displayed dense uniform dispersion in deionized water without conglomeration (Fig. 1a), which is necessary for making stable, void-free and gap-filling CeO_*x*_ thin films on flexible PEN substrates. However, the cross-linking of the organic ligands in the CeO_*x*_ sol–gel solution may hinder the charge transport and deteriorate the photovoltaic performance. With this in mind, we utilized a convenient UV–O_3_-processing approach to help the decomposition of organic ligands at a low temperature and reduce the spontaneous coalescence of the CeO_*x*_ nanocrystals (NCs) during preparation, thus producing high-quality ETLs. As presented in Fig. 1a, the urea-chelated CeO_*x*_ NCs self-organized into free-standing thin films by capillary-force-induced clustering of the dispersing medium during preparation.^33^ Under ozone and UV-light irradiation (UV–O_3_), oxygen-free radicals decomposed the urea ligands bound to the CeO_*x*_ NCs into NH_3_, CO_2_, and H_2_O. As the organic ligands decomposed, the CeO_*x*_ NCs would congregate on plastic substrate, finally leading to the direct attachment of the CeO_*x*_ film. Without the UV–O_3_ treatment, pinhole-free CeO_*x*_ thin films were successfully fabricated by the low-temperature reaction at 80 °C. In this paper, we made the first endeavor to create a dense CeO_*x*_ layer *via* ancillary ligand decomposition by applying UV–O_3_ without heat treatment. The crystal structures of the CeO_*x*_ NCs before and after the UV–O_3_ treatment were demonstrated by the X-ray diffraction (XRD) patterns. As shown in Fig. 1b, all the peaks in the XRD patterns of LT-CeO_*x*_ and UV-CeO_*x*_ were assignable to the cubic fluorite crystal structure (PDF no. 34-0394). We found that there was no significant difference in the microstructure between the CeO_*x*_ films obtained with and without UV–O_3_ treatment. As depicted in Fig. 1c, the decomposition of the organic ligands was demonstrated by the Fourier-transform infrared (FT-IR) spectra of the CeO_*x*_ films obtained with and without UV–O_3_ treatment. The bands around 3400 cm^−1^ and 1630 cm^−1^ could be ascribed to O–H stretching and bending vibrations, respectively, which are indicative of the remaining water molecules and the surface hydroxyl groups of the CeO_*x*_ thin films. The peak near 1127 cm^−1^ might be from the C–N stretching vibration of urea,^34^ while the peak at 1382 cm^−1^ was due to the deformation of ammonium produced by urea decomposition.^35^ Since both these peaks disappeared entirely after UV–O_3_ exposure for more than 30 min, urea was entirely decomposed by free radicals under UV irradiation. The band at 1496 cm^−1^ was ascribed to the stretching vibration of the carboxylates formed from urea decomposition.^36^ Lastly, the band near 480 cm^−1^ in the finger-print region corresponded to the vibrational stretching of the Ce–O bond formed under UV–O_3_ treatment. Furthermore, the removal of hydrophobic molecules achieved by this simple yet efficient method was confirmed by the improved wettability of the CeO_*x*_ film in hybrid perovskite solar cells because of the hydrophilic surface of CeO_*x*_ (Fig. S1 and S2†).

**Fig. 2:**
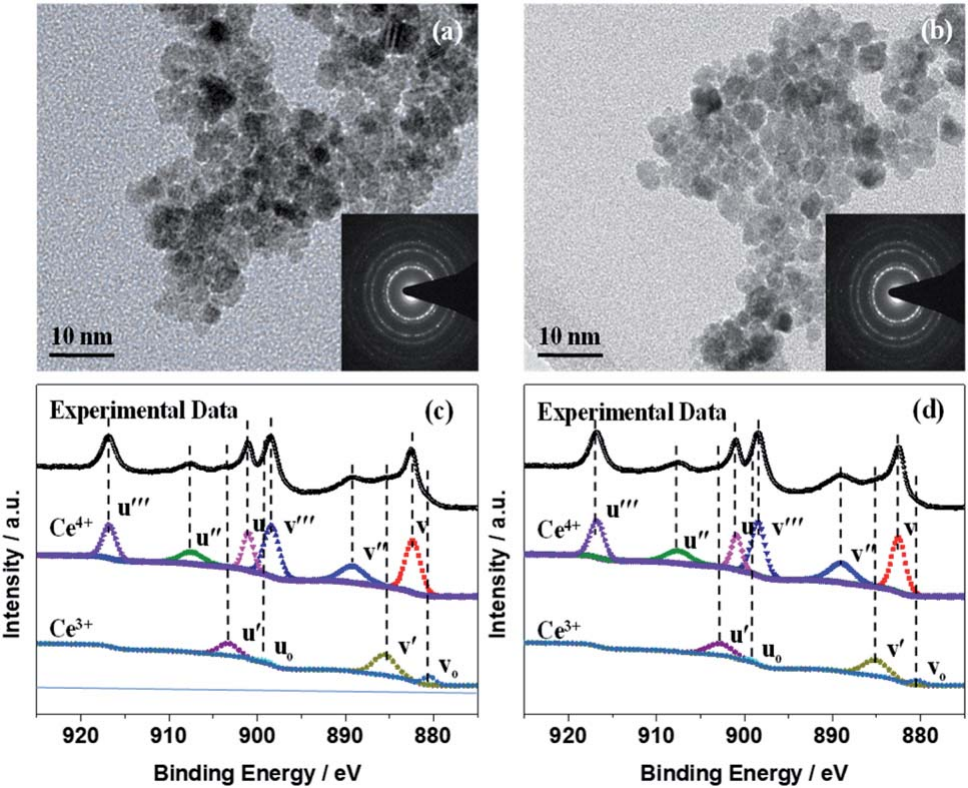
(a) TEM image before UV–O_3_ treatment. (b) TEM image after UV–O_3_ treatment. (c) XPS spectra before UV–O_3_ treatment. (d) XPS spectra after UV–O_3_ treatment. Insets of (a) and (b) are the corresponding SAEDs.

The transmission electron microscopy (TEM) images and diffraction patterns of CeO_*x*_ treated with UV–O_3_ (Fig. 2) demonstrated that the CeO_*x*_ nanoparticles consisted of relatively homogeneous crystalline phases. The uniform-sized dense bodies of the CeO_*x*_ NCs were *ca.* 6 nm, which is in close agreement with the particle size values obtained from the XRD analysis, as shown in Fig. 1b. The selected area electron diffraction (SAED) patterns (Fig. 2a and b, inset) indicated that the CeO_*x*_ particles were extremely crystalline with a cubic fluorite structure, which is consistent with the XRD patterns. The TEM images of the CeO_*x*_ material without (Fig. 2a) and with (Fig. 2b) UV–O_3_ treatment displayed no significant difference in morphology and crystal structure except for the larger particle size (*ca.* 10 nm) of the former.

X-ray photoelectron spectroscopy (XPS) measurements were used to confirm the composition of the CeO_*x*_ film, as depicted in Fig. 2c and d. The XPS survey scans (Fig. S4†) showed the elemental spectra of Ce, O, and C, and the adventitious C was used as the charge reference. According to an equation reported in the literature (Fig. S4†),^37,38^ the concentration of Ce^3+^ was deduced from the Ce 3d spectra of the CeO_*x*_ films obtained without and with UV–O_3_ treatment to be 0.27 and 0.21 (Fig. 2c and d), based on which the *x* values in the obtained CeO_*x*_ samples were calculated as 1.86 and 1.89, respectively. As far as variation in the elemental composition is concerned, the contents of Ce^3+^ and Ce^4+^ in the UV–O_3_ treated CeO_*x*_ film were similar to those in the film prepared without the treatment.

**Fig. 3:**
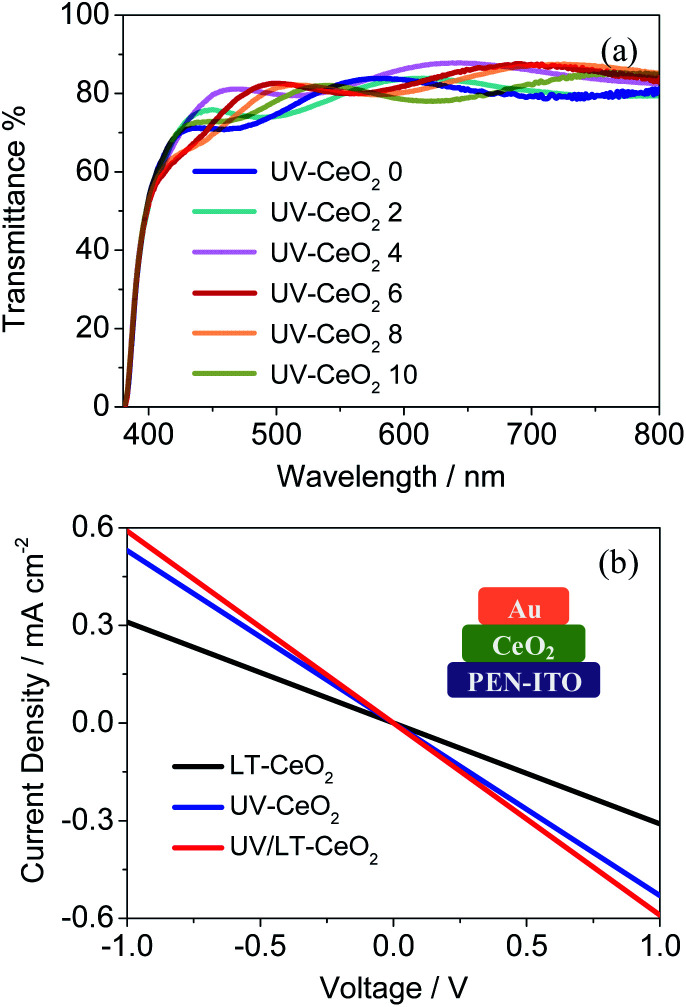
(a) Transmittance spectra of the CeO_*x*_ films with different thicknesses. The number after “CeO_*x*_” in the legend indicates the number of CeO_*x*_ ETLs spin-coated on the substrate. (b) Conductivity of the CeO_*x*_ films produced using different treatment methods.

The structural, optical and electrical properties of the CeO_*x*_ films were systematically studied to decide the aptness of CeO_*x*_ as an ETL in PSCs. In general, the UV–O_3_ treatment is vital to get rid of the remaining solvents and enhance the properties of the CeO_*x*_ films.^32,39^ The UV–O_3_-processed CeO_*x*_ ETLs showed higher transmittance from 400 to 800 nm. As depicted in Fig. 3a, the optical transmittance of the CeO_*x*_ ETLs gradually enhanced with an increase in thickness up to 6 spin-coating cycles (71 nm). This is likely owing to the reduction in the surface roughness of the CeO_*x*_ ETLs coated on the PEN/ITO substrates relative to that of the bare PEN/ITO substrate. The top-view scanning electron microscopy (SEM) images showed a homogeneous integrated dense film on the ITO/PEN substrate spin-coated with the CeO_*x*_ sol–gel, as seen in Fig. S3.† The compact pinhole-free CeO_*x*_ films were successfully fabricated by 6 cycles of spin-coating under a UV–O_3_ atmosphere, which was in favor of improving the electron transport ability and increasing the perovskite deposition quantity between particles in the mesoporous framework. The current density *versus* voltage (*J*–*V*) characteristics were analyzed to investigate the impact of the UV–O_3_ treatment on electrical conductivity. The electrical conductivity of the UV/LT-CeO_*x*_ film was 1.38 × 10^−4^ S cm^−1^, which was more than twice as that of the LT-CeO_*x*_ film (0.65 × 10^−4^ S cm^−1^), due to the increased carrier density caused by the UV–O_3_ treatment.^40,41^ Noticeably, the UV-CeO_*x*_ film (1.04 × 10^−4^ S cm^−1^) also exhibited higher conductivity than the LT-CeO_*x*_ film. This is because the UV–O_3_ treatment hastened the disintegration of the stable surface groups and improved the links between the CeO_*x*_ nanoparticles (Fig. 3b), as well as charge transport. Meanwhile, the UV–O_3_-induced epitaxial attachment of the CeO_*x*_ nanocrystals in our study was similar to that reported by Hiraide *et al.*^42^

**Fig. 4:**
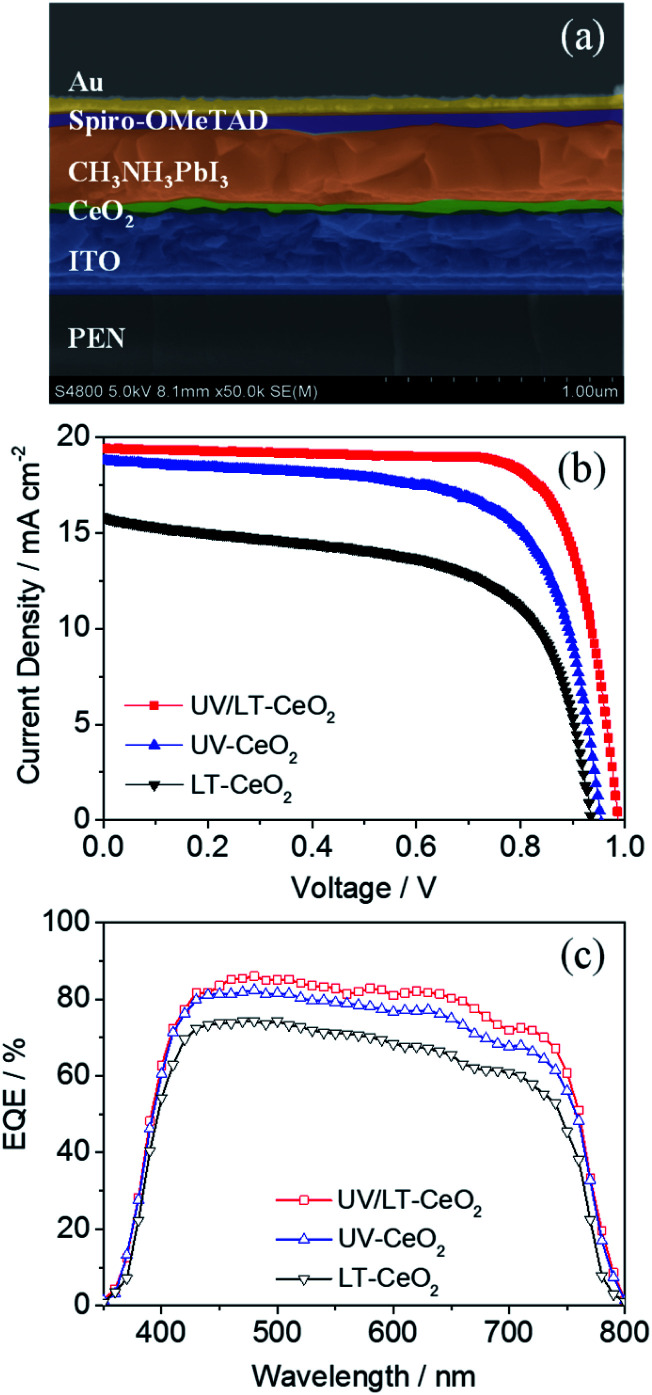
(a) Cross-sectional SEM image of the f-PSC based on the UV/LT-CeO_*x*_ ETL. (b) *J*–*V* curves and (c) IPCE spectra of the high-performing f-PSCs fabricated with the LT-CeO_*x*_, UV-CeO_*x*_, and UV/LT-CeO_*x*_ ETLs.

The relationship between the thickness of the CeO_*x*_ ETL and the photovoltaic performance is illustrated in Fig. S5.†*J*_sc_ and *V*_oc_ initially enhanced as the film thickness increased, while they decreased beyond a certain film thickness. This result showed that the generation of photocurrent in CeO_*x*_ PSCs would be dominated by high-quality ETLs below *ca.* 70 nm. To understand the electron transfer pathway in CeO_*x*_ ETL, ultraviolet photoelectron spectra (UPS) and UV-vis absorbance spectra were obtained to establish the energy levels (Fig. S6†). The architecture and energy-level diagram of the CeO_*x*_-based f-PSCs are illustrated in Fig. S7.† Therefore, planar f-PSCs based on the LT-CeO_*x*_, UV-CeO_*x*_ and UV/LT-CeO_*x*_ ETLs were fabricated to explore their photovoltaic performance. As we know, good-quality perovskite films are key to highly-efficient stable PSCs. We prepared good quality CH_3_NH_3_PbI_3_ layers on different ETLs by adopting the Lewis-base adduct approach,^43^ which showed no effect on the perovskite morphology. The cross-sectional SEM image in Fig. 4a shows the device composed of typical perovskite layers: FTO/UV-CeO_*x*_ ETL (70 nm)/CH_3_NH_3_PbI_3_ (480 nm)/spiro-OMeTAD (180 nm)/Au (88 nm). Fig. 4b exhibits the typical current density–voltage (*J*–*V*) curves of the cells built with the LT-CeO_*x*_, UV-CeO_*x*_, and UV/LT-CeO_*x*_ ETLs. The ETL dependence of various photovoltaic parameters of the solar cells is listed in Table 1. The devices based on the LT-CeO_*x*_ ETLs prepared at 80 °C exhibited a *J*_sc_ of 15.81 mA cm^−2^, a *V*_oc_ of 0.92 V, a fill factor (FF) of 0.62, and PCE of 9.08%. In contrast, the PSCs based on the UV-CeO_*x*_ ETLs exhibited distinctly advantageous performance with a *J*_sc_ of 18.78 mA cm^−2^, a *V*_oc_ of 0.94, an FF of 0.69, and PCE of 12.21%. When the CeO_*x*_ ETLs prepared at low temperature were irradiated by UV–O_3_ treatment, the corresponding UV/LT-CeO_*x*_-based PSCs showed remarkably higher characteristic factors with a *J*_sc_ of 19.42 mA cm^−2^, a *V*_oc_ of 0.98 V, an FF of 0.72, and the best PCE at 14.63%. The extensive performance statistics for *ca.* 75 devices in each group can be found in Fig. S8.† The reasons for the improved performance of the UV/LT-CeO_*x*_ devices than the LT-CeO_*x*_ ones are better transparency, advantageous electrochemical properties, and the higher hole-blocking effect, which may be due to the decreased charge recombination since the UV–O_3_ treatment optimizes the defects sites on the CeO_*x*_ film surface.^44,45^Fig. 4c illustrates the incident photon-to-current efficiency (IPCE) spectra of the cells. Compared with devices based on UV-CeO_*x*_ and LT-CeO_*x*_, the broad spectral response (300–760 nm) of the UV/LT-CeO_*x*_ devices exhibited a higher IPCE value dependence, which was devoted to the exceptional optical transparency and electrical conductivity of the CeO_*x*_ ETL. The ratio of the respective integrated IPCE spectra of each batch corresponded with the ratio of the photocurrents.

**Table 1:** Summary of the photovoltaic performance metrics of the best and average devices based on the UV/LT-CeO_*x*_, UV-CeO_*x*_, LT-CeO_*x*_ ETLs under AM 1.5 illumination (100 mW cm^−2^)^a^

Sample	*J*_sc_/mA cm^−2^	*V*_oc_/V	FF/%	PCE/%
LT-CeO_*x*-max_	15.81	0.92	62.19	9.08
LT-CeO_*x*-avg_	15.93	0.91	58.72	8.49
UV-CeO_*x*-max_	18.78	0.94	69.31	12.21
UV-CeO_*x*-avg_	17.75	0.93	69.89	11.56
UV/LT-CeO_*x*-max_	19.42	0.98	76.21	14.63
UV/LT-CeO_*x*-avg_	19.01	0.96	74.38	13.50

a Max and avg represent the best and the average devices, respectively.

**Fig. 5:**
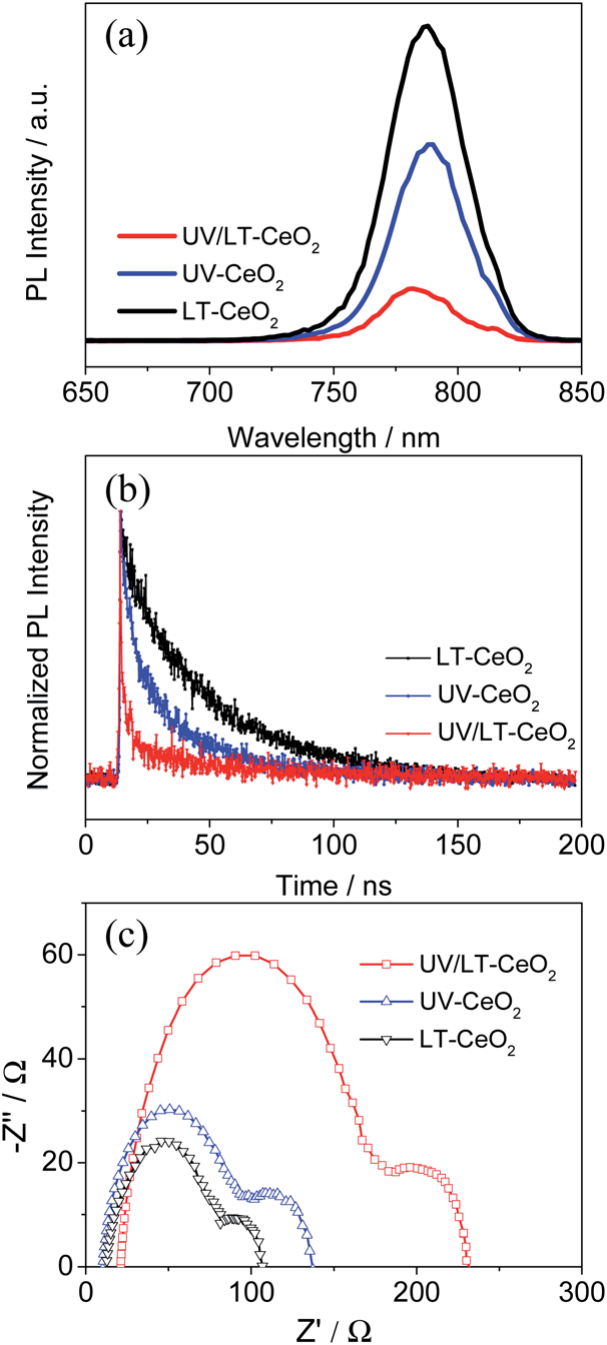
(a) Normalized steady-state photoluminescence (PL) spectra, (b) normalized time-resolved photoluminescence (TRPL) spectra and (c) EIS spectra of the LT-CeO_*x*_, UV-CeO_*x*_, and UV/LT-CeO_*x*_ ETLs in PSCs.

To investigate the effect of doping UV–O_3_ in detail, PL and TRPL spectroscopy were performed to probe the charge transfer process between the CeO_*x*_ ETL and the perovskite layer after UV–O_3_ doping. Fig. 5a exhibits that the quenching of the intrinsic CH_3_NH_3_PbI_3_ fluorescence emission by the ETLs occurred at the spectral peak (766 nm). Compared with the LT-CeO_*x*_/perovskite sample, the PL intensities of UV-CeO_*x*_/perovskite and UV/LT-CeO_*x*_/perovskite were drastically reduced with the UV–O_3_ doping process, indicating that the photocurrent density and recombination rate reduction were the most efficient in the UV–O_3_-processed ETLs. A higher increase in amplitude accompanied by a slow PL quenching, produced in a progressive manner from the perovskite and its interfaces. Therefore, the UV–O_3_ doping process is beneficial for speedy charge transport and enhanced charge generation and collection in the device. Fig. 5b presents the TRPL spectra and the matching PL decay times evaluated using an apparently bi-exponential decay model. The results for the LT-CeO_*x*_/perovskite, UV-CeO_*x*_/perovskite and UV/LT-CeO_*x*_/perovskite samples are summarized in Table S1.† The fast decay corresponded to the quenching of carriers from the perovskite layer to the ETLs, and the slow decay was regarded as the result of irradiative decay within the perovskite film.^31,46,47^ In comparison with the LT-CeO_*x*_ film, the UV-CeO_*x*_ ETL-based devices exhibited a fast decay constant decay, which dominated the charge extraction from the perovskite to the ETLs. The average lifetime at the interface between LT-CeO_*x*_ and the perovskite film was 31.20 ns, which severely decreased to 26.11 ns at the interface between UV-CeO_*x*_ and the perovskite film. In comparison with UV-CeO_*x*_/perovskite, a faster decay component could be observed at the UV/LT-CeO_*x*_/perovskite interface under increased amplitude (2.68 ns to 1.80 ns), which induced a low average lifetime (18.21 ns). Thus, the UPS spectra (Fig. S6b†) and PL emission spectra exhibited that charge transport and extraction were strongly enhanced by the synergistic effects of the sharp-shift in the energy level and the raised electrical conductivity, resulting in great enhancement of the photocurrent efficiency of the UV/LT-CeO_*x*_ ETL.

To further comprehend the improvement in performance upon UV–O_3_ treatment, electrochemical impedance spectroscopy (EIS) measurements were carried out on different devices since it is considered as an effective method to investigate the carrier transport behavior and interfacial properties of PSCs.^48,49^ The Nyquist plots displayed in Fig. 5c were obtained under dark at an applied bias voltage of 0.7 V in the frequency range from 0.1 to 100 kHz, in which two different semicircles are shown in the high- and low-frequency ranges. The Nyquist plots were fitted with an equivalent circuit containing a series resistance (*R*_s_) in series with two RC elements. The fitted transport resistance (*R*_co_) and recombination resistance (*R*_rec_) were plotted in connection with UV–O_3_ and thermal treatments, as illustrated in Fig. S9 and Table S2.†^50,51^ When the CeO_*x*_ film was treated at 80 °C, the water molecules in the ETLs could not be effectively removed to obtain ideal transmission resistance (LT-CeO_*x*_: *R*_co_ = 116.5 Ω cm^2^). In contrast, upon UV–O_3_ treatment, the *R*_co_ decreased significantly (*R*_co_ = 102.4 Ω cm^2^ for the UV-CeO_*x*_ film and 87.8 Ω cm^2^ for the UV/LT-CeO_*x*_ film), which is in accordance with the conductivity measurements (Fig. 3b). Moreover, compared with the *R*_rec_ of the LT-CeO_*x*_-based interfaces (336.2 Ω cm^2^), the *R*_rec_ of the UV-CeO_*x*_-based interfaces had increased to 456.9 Ω cm^2^, and that of the UV/LT-CeO_*x*_-based interfaces increased to 573.6 Ω cm^2^. Undoubtedly, the UV–O_3_ treatment optimized the CeO_*x*_/perovskite interface for a large part and reduced the recombination rate significantly by increasing the *R*_rec_. Therefore, UV–O_3_ treatment is conducive to realizing outstanding low-temperature CeO_*x*_ ETLs and achieving high-performance perovskite devices.

**Fig. 6:**
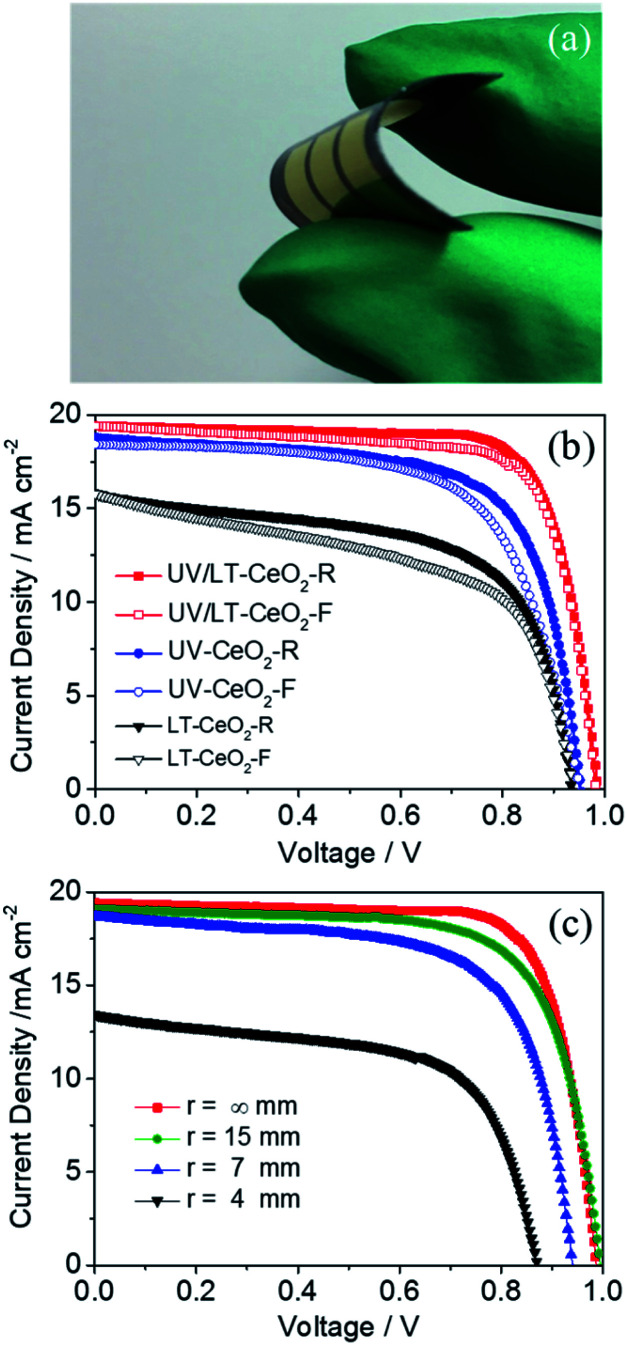
(a) Photograph of the f-PSC. (b) Hysteresis behavior of the f-PSCs based on the LT-CeO_*x*_, UV-CeO_*x*_, and UV/LT-CeO_*x*_ ETLs, where *F* indicates the forward scan and *R* indicates the reverse scan in the *J*–*V* curves. (c) *J*–*V* curves of the f-PSCs with various radii (*r*) after recovery from 500 bending cycles.

The low-temperature-processed ETLs subjected to the UV process are highly desirable for constructing high-performance flexible PSCs. Highly densely-packed CeO_*x*_ ETLs were effectively prepared on sputtered PEN/ITO substrates, and then typical devices with the ETL/CH_3_NH_3_PbI_3_/spiro-OMeTAD/Au configuration were consecutively fabricated by conventional methods. The photograph of the flexible CeO_*x*_-based PSC with UV–O_3_ treatment is shown in Fig. 6a. The hysteresis effect under different *J*–*V* scans directions or rates were exhibited in PSCs, especially n–i–p planar devices.^52,53^ It has been proposed in previous works that the hysteresis initiates from polarization caused by ferroelectricity, ion motion of perovskite materials, and an unbalanced flux carrier density.^54,55^ As shown in Fig. 6b, we discovered that the devices based on the LT-CeO_*x*_ ETL showed large hysteresis between the forward and reverse scanning directions. Nevertheless, this hysteresis behavior was greatly lessened in the UV-CeO_*x*_ ETL devices, and the UV/LT-CeO_*x*_ ETL-based ones illustrated the smallest hysteresis among the devices. This phenomenon most likely stemmed from the reduced accumulation of interfacial charge in the UV/LT-CeO_*x*_/perovskite interface. This result is consistent with the current hysteresis and photovoltaic performance. That is, the higher the charge extraction efficiency of selective contact, the lesser is the current hysteresis in the *J*–*V* curves, and the higher is the photovoltaic performance of the f-PSC. The stabilized photocurrents of the devices at 0.88 V bias under 1 sun illumination are tracked in Fig. S10a,† and the stabilized PCEs at the different bending radii of 15, 7 and 4 mm reached 13.96, 11.95 and 10.71% at 200 s continuous irradiation, respectively, as indicated in Fig. S10b.† To assess the stability of the devices with and without UV–O_3_ treatment, the long-term stability of cells stored in the air with 10% RH at 25 °C without packing was compared. The PCEs of the devices fabricated with UV/LT, UV and LT ETLs were measured continuously for 550 h. As presented in Fig. S10c,† the half-life of the device treated with UV/LT was at least 200 h longer than the other two half-life periods. This might be due to the compact perovskite film with a low trap-state density, which could effectively resist moisture penetration. Fig. 6c shows the *J*–*V* curves of the f-PSC as a function of the bending curvature radius and bending cycles. The remarkable PCE of the f-PSC was 14.63%. After 500 bending cycles at *r* = 15, 7 and 4 mm, the PCE values were 13.27%, 8.53% and 4.41%, respectively. These results illustrated that the initial efficiency was reduced to *ca.* 90% at *r* = 15 mm, but was caused devastating at *r* = 4 mm. We can assume that the slight decrease in PCE at *r* = 15 mm was due to the plastic deformation of the ITO/PEN substrate. However, the brittle ITO could possibly have broken when bent at *r* = 4 mm, which is consistent with previous reports.^56,57^ Meanwhile, the photovoltaic parameters (*J*_sc_, *V*_oc_, and FF) showed an almost similar decreased tendency with a 2% loss after 500 bending cycles. Hence, we could conclude that the structure of the cell with *r* = 15 mm possessed outstanding mechanical durability. The durability test was also performed on the three devices at *r* = 15, 7 and 4 mm, for up to 500 cycles (Fig. S10d†). When the device was deformed once, the device performance decreased linearly at the same bending radius. The cells bent at 15 mm showed excellent stability with less than 10% reduction of the PCE after repeated bending at ambient temperature.

## 4. Conclusions

In this work, a low-temperature process (80 °C) for preparing highly efficient f-PSCs involving UV–O_3_ treatment of ligand-capped CeO_*x*_ NCs was developed. The photocatalytic decomposition of the organic ligands accompanied the spontaneous coalescence of the NCs, resulting in highly efficient ETLs, which exhibited high performance, including high film compactness, high electrical conductivity, and high transparency, as well as favorable energy levels matching with the perovskite. Using the UV–O_3_ treated CeO_*x*_ ETLs prepared at a low temperature, highly efficient planar PSCs with the best PCE of 14.63% was achieved with ITO/PEN substrates. We report a facile route that is very appropriate for the construction of high-performance f-PSCs because it easily modifies the ETLs at a low-temperature. This approach will pave the way for further development of f-PSCs and is feasible for large-scale roll-to-roll processes.

## Conflicts of interest

The authors declare no competing financial and nonfinancial interests.

## Supplementary Material

NA-002-D0NA00367K-s001

## References

[cit1] Burschka J., Pellet N., Moon S. J., Baker R. H., Gao P., Nazeeruddin M. K., Grätzel M. (2013). Nature.

[cit2] Turren-Cruz S. H., Hagfeldt A., Saliba M. (2018). Science.

[cit3] Ru P. B., Bi E. B., Zhang Y., Wang Y. B., Kong W. Y., Sha Y. M., Tang W. T., Zhang P., Wu Y. Z., Chen W., Yang X. D., Chen H., Han L. Y. (2020). Adv. Energy Mater..

[cit4] Wu C. C., Wang D., Zhang Y. Q., Gu F. D., Liu G. H., Zhu N., Luo W., Han D., Guo X., Qu B., Wang S. F., Bian Z. Q., Chen Z. J., Xiao L. X. (2019). Adv. Funct. Mater..

[cit5] Huang K. Q., Peng Y. Y., Gao Y. X., Shi J., Li H. Y., Mo X. D., Huang H., Gao Y. L., Ding L. M., Yang J. L. (2019). Adv. Energy Mater..

[cit6] Lee M. M., Teuscher J., Miyasaka T., Murakami T. N., Snaith H. J. (2012). Science.

[cit7] Yang W. S., Noh J. H., Jeon N. J., Kim Y. C., Ryu S., Seo J., Seok S. I. (2015). Science.

[cit8] Giacomo F. D., Zardetto V., D'Epifanio A., Pescetelli S., Matteocci F., Razza S., Carlo A. D., Licoccia S., Kessels W. M. M., Creatore M., Brown T. M. (2015). Adv. Energy Mater..

[cit9] Jeong I., Jung H., Park M., Park J. S., Son H. J., Joo J., Lee J., Ko M. J. (2016). Nano Energy.

[cit10] Ryu U., Jee S., Park J., Han K., Lee J. H., Park M., Choi K. M. (2018). ACS Nano.

[cit11] Lee M., Jo Y., Kim D. S., Jeong H. Y., Jun Y. (2015). J. Mater. Chem. A.

[cit12] Tao C., Neutzner S., Colella L., Marras S., Kandada A. R. S., Gandini M., Bastiani M. D., Pace G., Manna L., Caironi M., Bertarelli C., Petrozza A. (2015). Energy Environ. Sci..

[cit13] Leijtens T., Eperon G. E., Pathak S., Abate A., Lee M. M., Snaith H. J. (2013). Nat. Commun..

[cit14] Li W., Zhang W., Reenen S. V., Sutton R. J., Fan J., Haghighirad A. A., Johnston M. B., Wang L., Snaith H. J. (2016). Energy Environ. Sci..

[cit15] Boix P. P., Nonomura K., Mathews N., Mhaisalkar S. G. (2014). Mater. Today.

[cit16] Bi D. Q., Boschloo G., Schwarzmuller S., Yang L., Johansson E. M. J., Hagfeldt A. (2013). Nanoscale.

[cit17] Hwang K., Jung Y. S., Heo Y. J., Scholes F. H., Watkins S. E., Subbiah J., Jones D. J., Kim D. Y., Vak D. (2015). Adv. Mater..

[cit18] Yang Y., You J., Hong Z., Chen Q., Cai M., Song T. B., Chen C. C., Lu S., Liu Y., Zhou H. (2014). ACS Nano.

[cit19] Heo J. H., Lee M. H., Han H. J., Patil B. R., Yu J. S., Im S. H. (2016). J. Mater. Chem. A.

[cit20] Yang J., Siempelkamp B. D., Mosconi E., Angelis F. D., Kelly T. L. (2015). Chem. Mater..

[cit21] Cheng Y., Yang Q. D., Xiao J., Xue Q., Li H. W., Guan Z., Yip H. L., Tsang S. W. (2015). ACS Appl. Mater. Interfaces.

[cit22] Wali Q., Fakharuddin A., Ahmed I., Ab Rahim M. H., Ismail J., Jose R. (2014). J. Mater. Chem. A.

[cit23] Jiang Q., Zhang L., Wang H., Yang X., Meng J., Liu H., Yin Z., Wu J., Zhang X., You J. (2016). Nat. Energy.

[cit24] Ke W., Fang G., Liu Q., Xiong L., Qin P., Tao H., Wang J., Lei H., Li B., Wan J., Yang G., Yan Y. (2015). J. Am. Chem. Soc..

[cit25] Correa Baena J. P., Steier L., Tress W., Saliba M., Neutzner S., Matsui T., Giordano F., Jacobsson T. J., Srimath Kandada A. R., Zakeeruddin S. M., Petrozza A., Abate A., Nazeeruddin M. K., Grätzel M., Hagfeldt A. (2015). Energy Environ. Sci..

[cit26] Wang C., Zhao D., Grice C. R., Liao W., Yu Y., Cimaroli A., Shrestha N., Roland P. J., Chen J., Yu Z., Liu P., Cheng N., Ellingson R. J., Zhao X., Yan Y. (2016). J. Mater. Chem. A.

[cit27] Corma A., Atienzar P., García H., Ching J. Y. C. (2004). Nat. Mater..

[cit28] Jose R., Thavasi V., Ramakrishna S. (2009). J. Am. Ceram. Soc..

[cit29] Wang X., Deng L. L., Wang L. Y., Dai S. M., Xing Z., Zhan X. X., Lu X. Z., Xie S. Y., Huang R. B., Zheng L. S. (2017). J. Mater. Chem. A.

[cit30] Hu T., Xiao S. Q., Yang H. J., Chen L., Chen Y. W. (2018). Chem. Commun..

[cit31] Periyat P., Laffir F., Tofaila S. A. M., Magner E. (2011). RSC Adv..

[cit32] Pang A. Y., Shen D. L., Wei M. D., Chen Z. N. (2018). ChemSusChem.

[cit33] Pillai S. C., Periyat P., Kutty R. G., McCormack D. E., Seery M. K., Hayden H., Colreavy J., Corr D., Hinder S. J. (2007). J. Phys. Chem. C.

[cit34] Ren L., Huang X., Sun F., He X. (2007). Mater. Lett..

[cit35] Gu H., Soucek M. D. (2007). Chem. Mater..

[cit36] Tan Z. A., Li L., Li C., Yan L., Wang F., Xu J., Yu L., Song B., Hou J., Li Y. (2014). Adv. Mater. Interfaces.

[cit37] Watanabe S., Ma X., Song C. (2009). J. Phys. Chem. C.

[cit38] Jung K. H., Seo J. Y., Lee S., Shin H., Park N. G. (2017). J. Mater. Chem. A.

[cit39] Ke W., Zhao D., Cimaroli A. J., Grice C. R., Qin P., Liu Q., Xiong L., Yan Y., Fang G. (2015). J. Mater. Chem. A.

[cit40] Nikolay T., Larina L., Shevaleevskiy O., Ahn B. T. (2011). Energy Environ. Sci..

[cit41] Chen W., Wu Y., Yue Y., Liu J., Zhang W., Yang X., Chen H., Bi E., Ashraful I., Grätzel M., Han L. (2015). Science.

[cit42] Hiraide T., Kageyama H., Nakagawa Y., Oaki Y., Imai H. (2016). Chem. Commun..

[cit43] Ahn N., Son D. Y., Jang I. H., Kang S. M., Choi M., Park N. G. (2015). J. Am. Chem. Soc..

[cit44] Kim D. H., Han G. S., Seong W. M., Lee J. W., Kim B. J., Park N. G., Hong K. S., Lee S., Jung H. S. (2015). ChemSusChem.

[cit45] Lü X., Mou X., Wu J., Zhang D., Zhang L., Huang F., Xu F., Huang S. (2010). Adv. Funct. Mater..

[cit46] Niu G., Li W., Meng F., Wang L., Dong H., Qiu Y. (2014). J. Mater. Chem. A.

[cit47] Duan B., Ren Y. K., Xu Y. F., Chen W. Y., Ye Q., Huang Y., Zhu J., Dai S. Y. (2017). Inorg. Chem. Front..

[cit48] Bu T., Wu L., Liu X., Yang X., Zhou P., Yu X., Qin T., Shi J., Wang S., Li S., Ku Z., Peng Y., Huang F., Meng Q., Cheng Y. B., Zhong J. (2017). Adv. Energy Mater..

[cit49] Zarazua I., Han G., Boix P. P., Mhaisalkar S., FabregatSantiago F., Mora-Sero I., Bisquert J., Garcia-Belmonte G. (2016). J. Phys. Chem. Lett..

[cit50] Dagar J., Castro-Hermosa S., Gasbarri M., Palma A. L., Cina L., Matteocci F., Calabro E., Carlo A. D., Brown T. M. (2018). Nano Res..

[cit51] Bu T., Shi S. W., Li J., Liu Y. F., Shi J. L., Chen L., Liu X. P., Qiu J. H., Ku Z. L., Peng Y., Zhong J., Cheng Y. B., Huang F. Z. (2018). ACS Appl. Mater. Interfaces.

[cit52] Snaith H. J., Abate A., Ball J. M., Eperon G. E., Leijtens T., Noel N. K., Stranks S. D., Wang J. T. W., Wojciechowski K., Zhang W. (2014). J. Phys. Chem. Lett..

[cit53] Heo J. H., Han H. J., Kim D., Ahn T. K., Im S. H. (2015). Energy Environ. Sci..

[cit54] Shao Y., Xiao Z., Bi C., Yuan Y., Huang J. (2014). Nat. Commun..

[cit55] Li C., Tscheuschner S., Paulus F., Hopkinson P. E., Kießling J., Köhler A., Vaynzof Y., Huettner S. (2016). Adv. Mater..

[cit56] Kim B. J., Kim D. H., Lee Y. Y., Shin H. W., Han G. S., Hong J. S., Mahmood K., Ahn T. K., Joo Y. C., Hong K. S., Park N. G., Lee S., Jung H. S. (2015). Energy Environ. Sci..

[cit57] Jo J. W., Seo M. S., Park M., Kim J. Y., Park J. S., Han I. K., Ahn H., Jung J. W., Sohn B. H., Ko M. J., Son H. J. (2016). Adv. Funct. Mater..

