# Supplementary material for: UV–O3 treated annealing-free cerium oxide as electron transport layers in flexible planar perovskite solar cells
Source: Nanoscale Adv. 2020 Jul 23;2(9):4062–9. doi: 10.1039/d0na00367k (PMC9419069; doi:10.1039/d0na00367k)
Supplement: NA-002-D0NA00367K-s001 [file NA-002-D0NA00367K-s001.pdf]

Electronic Supplementary Material (ESI) for Nanoscale Advances.
This journal is © The Royal Society of Chemistry 2020

Electronic Supplementary Information (ESI)

UV-O_3 Treated and Annealing-free Cerium Oxide as Electron Transport Layers for Flexible Planar Perovskite Solar Cells

Aiying Pang,[a,b] *Jinlong Li,* [a,c] *Xiao-Feng Wei,*[d] *Zhi-Wu Ruan,*[a,b] *Ming Yang*[a,b] *and Zhong-Ning Chen*[a, b*]

a *State Key Laboratory of Structural Chemistry, Fujian Institute of Research on the Structure of Matter, Chinese Academy of Sciences, Fuzhou, Fujian 350002, China*
b *College of Chemistry and Materials, Fujian Normal University, Fuzhou, Fujian 350007, China*
c College of Chemistry, Fuzhou University, Fuzhou, Fujian 350002, China
d *National Engineering Research Center of Chemical Fertilizer Catalyst, School of Chemical Engineering, Fuzhou University, Fujian, 350002, China*

E-mail: *czn@fjirsm.ac.cn*

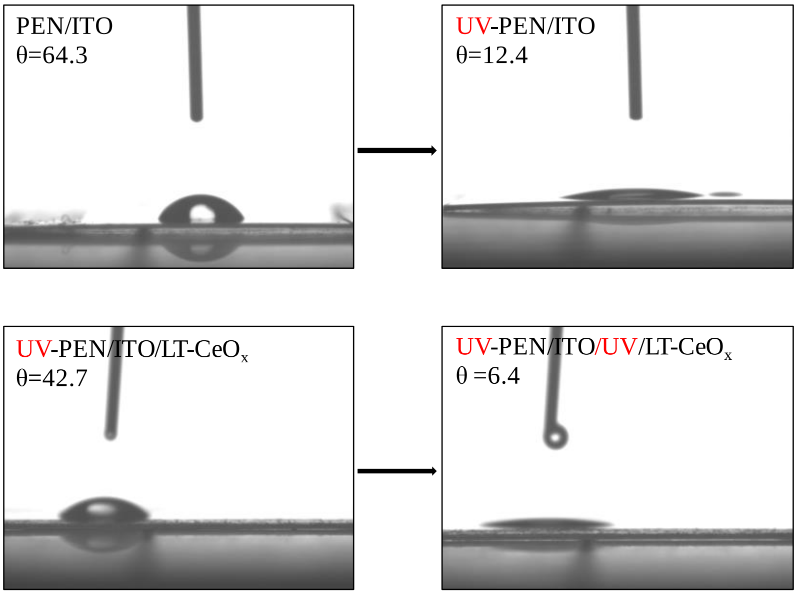

Fig. S1 The contact angles of substrate of PEN/ITO and aqueous-processed CeO_x sol-gel droplet before and after UV-O_3 processing.

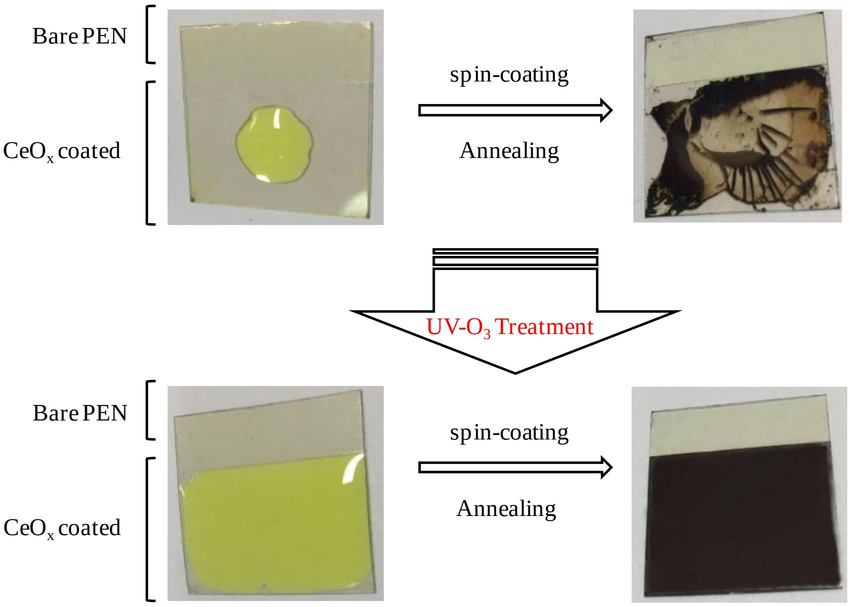

Fig. S2 Photographs of the deposition of the perovskite solution and spin-coated perovskite film on CeO_x layers before (top) and after (bottom) UV-O_3 treatment.

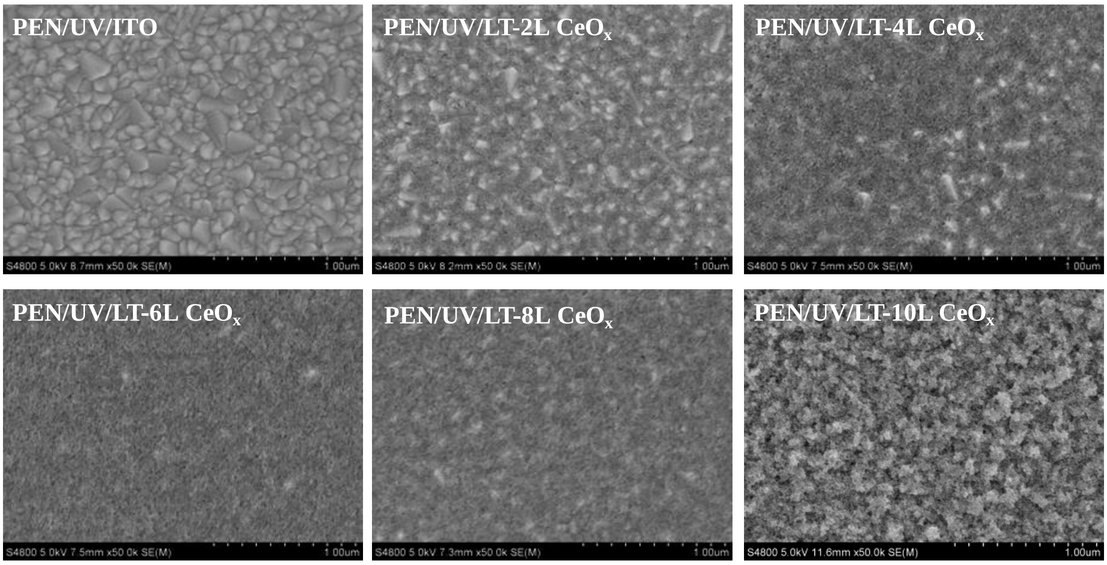

Fig. S3 SEM images of CeO_x thin films on PEN/ITO substrate with a different thickness. The “nL” before “CeO_x” indicates the number of CeO_x sol-gel spin-coated on the substrate.

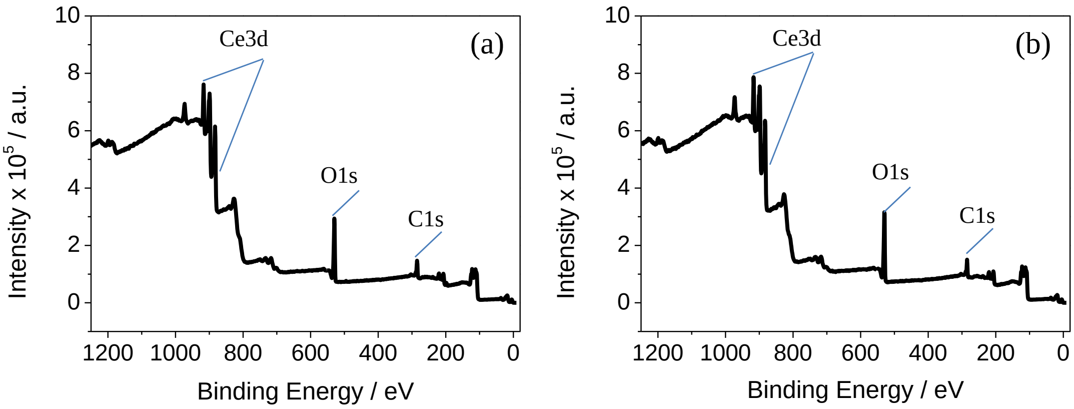

$$C_{Ce^{3+}} = \frac{\nu_0 + \nu' + \upsilon_0 + \upsilon'}{\sum(\nu + \upsilon)} \qquad (c)$$

Fig. S4 XPS survey scan of CeO_x film spin-coated on FTO substrate before (a) and after (b) UV-O_3 treatment. (c) The formula for calculating Ce^{3+}contentequation in CeO_x.

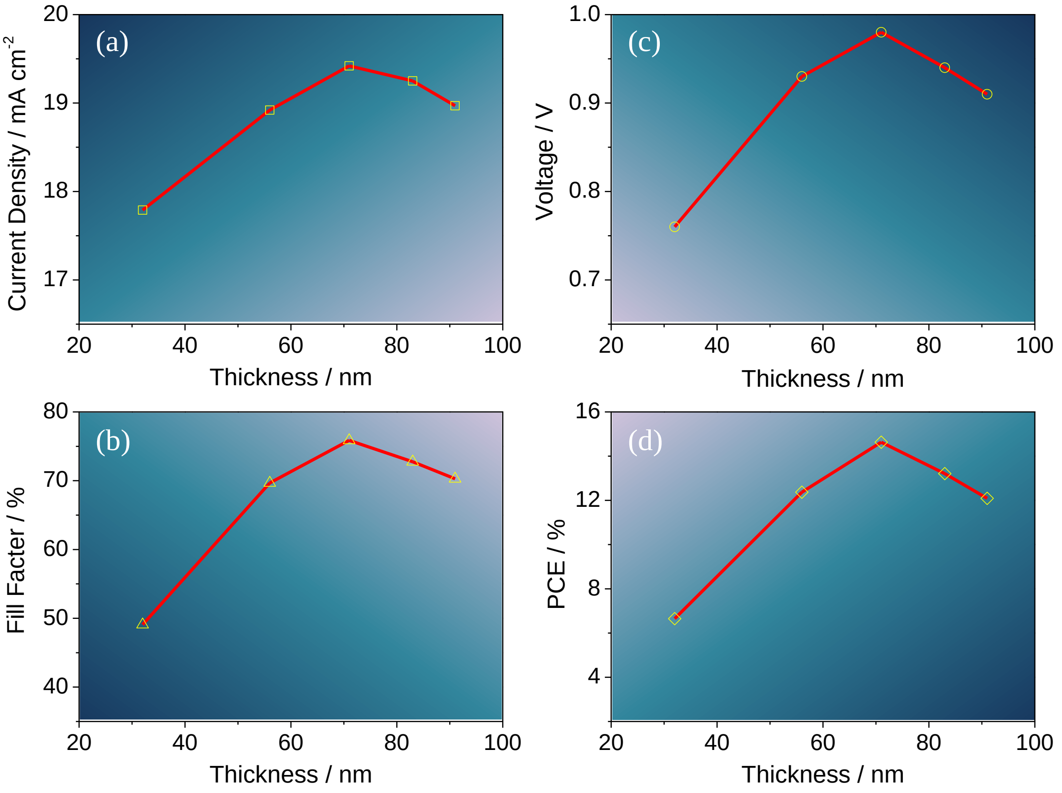

Fig. S5 The dependence of photovoltaic performances on film thickness: (a) J_{sc}, (b) V_{oc}, (c) *FF* and (c) PCE (1 Sun, 100 mW cm^{-2}, opened cells).

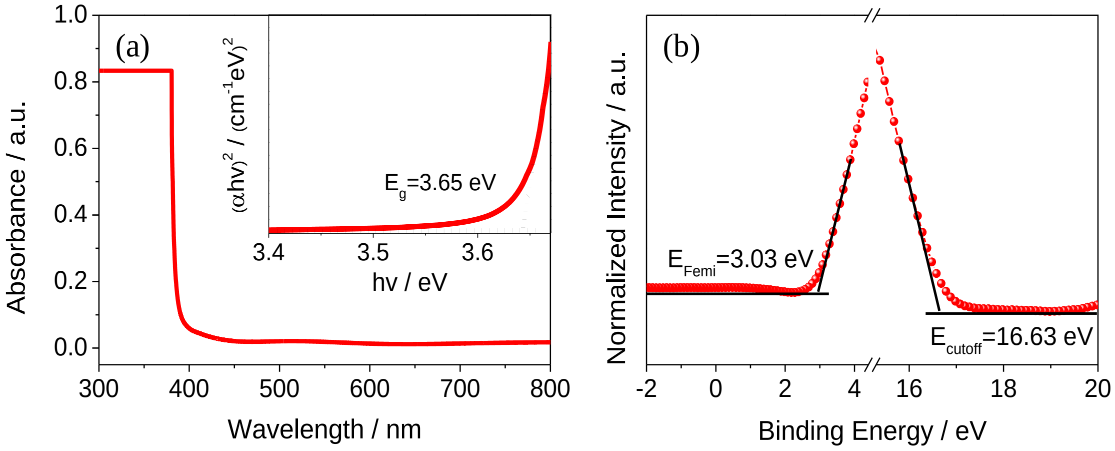

Fig. S6 (a) UV-vis absorbance spectra (the inset is plots of $(\alpha hv)^2$ versus energy) of CeO_x and (b) UPS spectra of the CeO_x film.

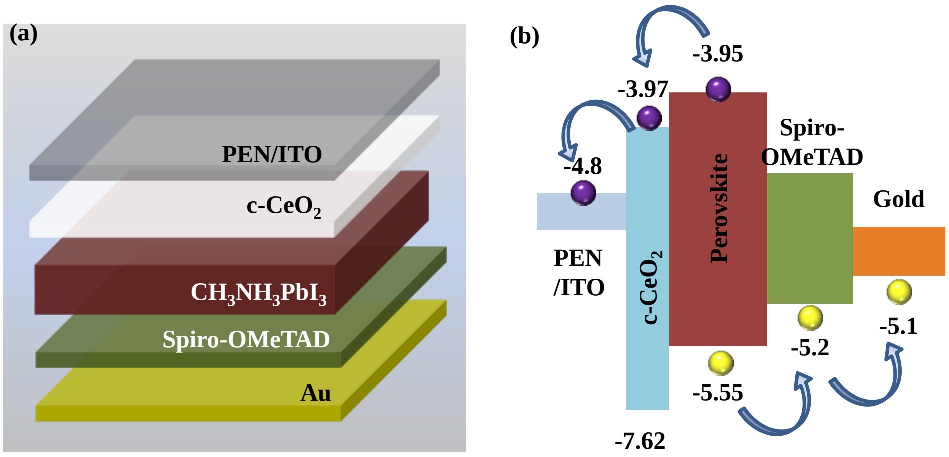

Fig. S7 (a) A schematic view of the device structure. (b) An energy band diagram of the various device layers.

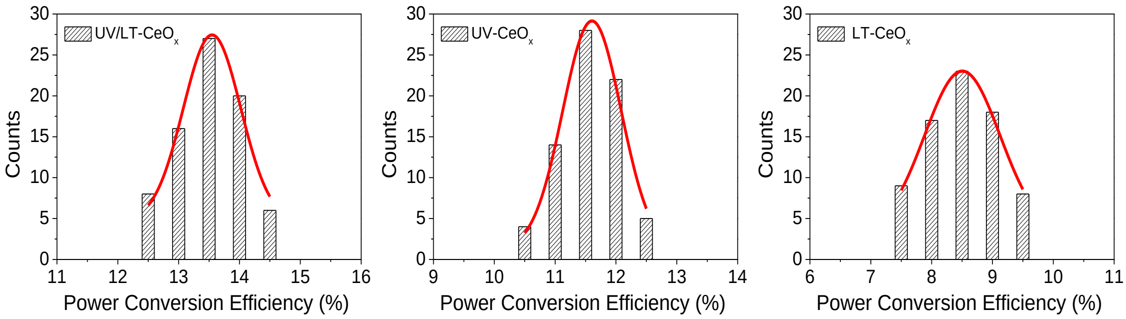

Fig. S8 Statistical histogram of PCE based on UV/LT-CeO$_x$, UV -CeO$_x$, LT-CeO$_x$ ETLs for ca.75 devices, fitted with a Gaussian distribution (red line).

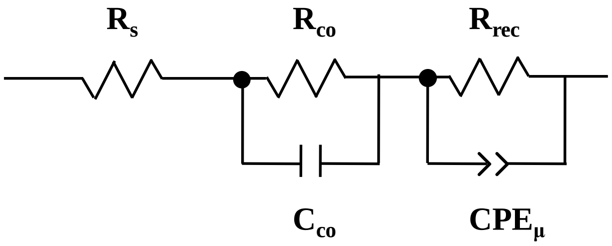

Fig. S9 The Nyquist plots were fitted by using an equivalent circuit mode.

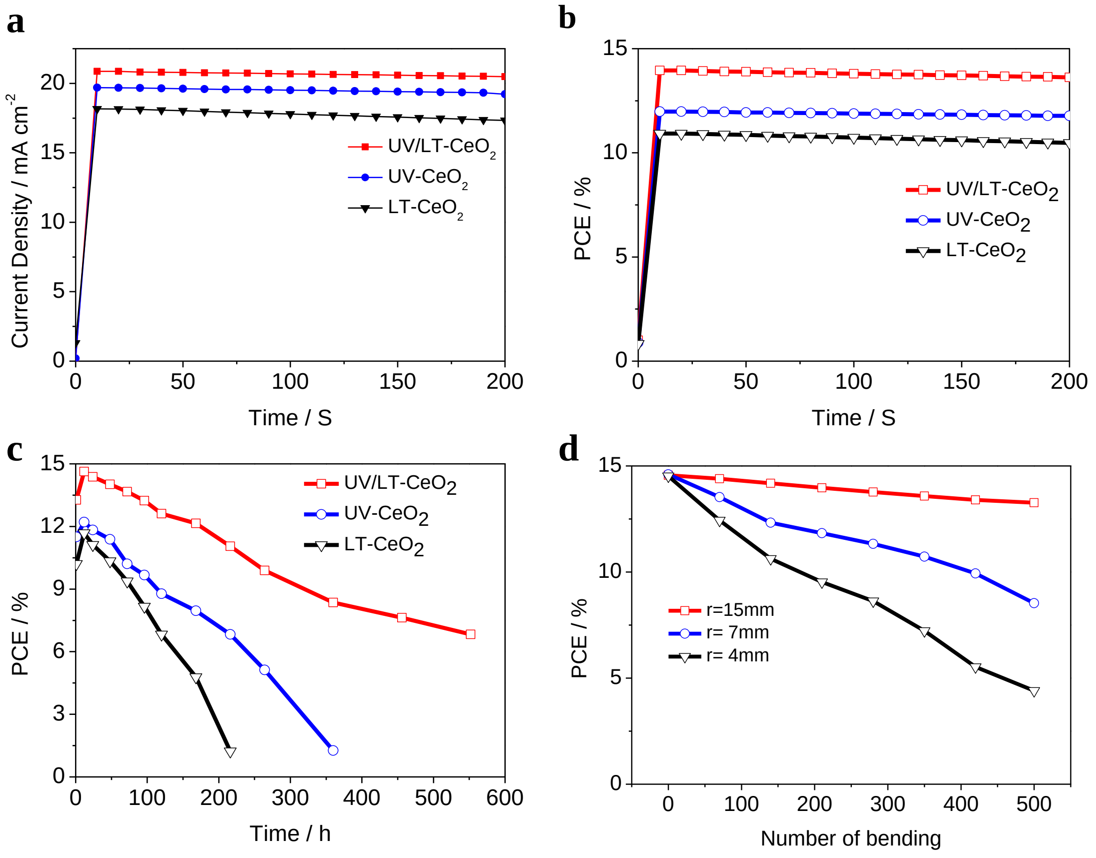

Fig. S10 (a) Stabilized photocurrent and (b) PCE at 0.88 V bias under 1 sun illumination. (c) Bending test of the devices for r = 15, 8 and 4 mm up to 500 bending cycles. (d) The long-term stability of f-PSCs stored in dry air with humidity of 10% RH at 25 °C without packed.

Table S1. Summary of the time-resolved photoluminescence (TRPL) spectra of the LT-CeO_x, UV-CeO_x, and UV/LT-CeO_x ETLs of PSCs.

Film	A_1	τ_1 /ns	A_2	τ_2 /ns	τ_{avg} /ns
UV/LT-CeO_x	86.5%	1.80±0.2	13.5%	123.4±4.8	18.21
UV-CeO_x	74.5%	2.68±0.3	25.5%	94.6±3.4	26.11
LT-CeO_x	63.9%	3.87±0.2	36.1%	79.6±5.7	31.20

Table S2. Deconvoluted resistances from impedance analyses for UV/LT-CeO_x, UV-CeO_x, LT-CeO_x ETLs.

ETL	R_s / Ω·cm^2	R_{co} / Ω·cm^2	R_{rec} / Ω·cm^2
LT-CeO_x	4.3	116.5	336.2
UV-CeO_x	3.9	102.4	456.9
UV/LT-CeO_x	1.9	87.8	573.6
